# Alteration in the mRNA expression of genes associated with gastrointestinal permeability and ileal TNF-α secretion due to the exposure of silver nanoparticles in Sprague–Dawley rats

**DOI:** 10.1186/s12951-019-0499-6

**Published:** 2019-05-13

**Authors:** Sarah E. Orr, Kuppan Gokulan, Mary Boudreau, Carl E. Cerniglia, Sangeeta Khare

**Affiliations:** 1 Division of Microbiology, National Center for Toxicological Research, US Food and Drug Administration, 3900 NCTR Road, Jefferson, AR 72029 USA; 2 Division of Biochemical Toxicology, National Center for Toxicological Research, US Food and Drug Administration, 3900 NCTR Road, Jefferson, AR 72029 USA

**Keywords:** Silver nanoparticles, Gastrointestinal toxicity, Intestinal permeability, Claudin, Tight junctions

## Abstract

**Background:**

Silver ions from silver nanoparticles (AgNP) or AgNPs themselves itself that are ingested from consumer health care products or indirectly from absorbed food contact material can interact with the gastrointestinal tract (GIT). The permeability of the GIT is strictly regulated to maintain barrier function and proper nutrient absorption. The single layer intestinal epithelium adheres and communicates actively to neighboring cells and the extracellular matrix through different cell junctions. In the current study, we hypothesized that oral exposure to AgNPs may alter the intestinal permeability and expression of genes controlling cell junctions. Changes in cell junction gene expression in the ileum of male and female rats administered different sizes of AgNP for 13-weeks were assessed using qPCR.

**Results:**

The results of this study indicate that AgNPs have an altering effect on cell junctions that are known to dictate intestinal permeability. mRNA expression of genes representing tight junction (*Cldn1, Cldn5, Cldn6*, *Cldn10* and *Pecam1*), focal adhesion (*Cav1, Cav2*, and *Itgb2*), adherens junction (*Pvrl1, Notch1*, and *Notch2*), and hemidesmosome (*Dst*) groups were upregulated significantly in females treated with 10 nm AgNP, while no change or downregulation of same genes was detected in male animals. In addition, a higher concentration of pro-inflammatory cytokine, TNF-α, was noticed in AgNP-treated female animals as compared to controls.

**Conclusions:**

This study proposes that interaction of silver with GIT could potentially initiate an inflammatory process that could lead to changes in the gastrointestinal permeability and/or nutrient deficiencies in sex-specific manner. Fully understanding the mechanistic consequences of oral AgNP exposure may lead to stricter regulation for the commercial usage of AgNPs and/or improved clinical therapy in the future.

## Introduction

Silver nanoparticles (AgNPs) are small, spherical particles of silver that range between 1 and 100 nm in size and continually release silver ions [1]. Nanoparticles can behave differently than larger particles of the same matter due to their extraordinary surface area to volume ratio [2, 3]. Use of AgNP is not currently authorized in the US; however, AgNPs have been incorporated into a variety of consumer goods worldwide including clothing, medical products, and food packaging as antimicrobials [4–6]. AgNPs have the unique property to prevent the growth of bacteria and viruses and are known to extend the shelf-life of many food products [7–10]. Silver ions from the AgNPs that are incorporated into food contact materials are likely to migrate into the food by diffusion, dissolution, and/or desorption [11, 12]. In addition, colloidal silver with AgNPs are found in health supplements sold commonly in stores that claim to support health [13, 14]. Inclusion of AgNP in consumer use products and health supplements prompted a need for the safety of such materials. In a 14-day monitored human oral dosing study two doses (10- and 32-ppm) of a commercial silver nanoparticle solution were consumed by healthy individuals over 14 days [15]. The results from this human study did not show observable clinically important toxicity markers. However, peak serum silver concentration was detected in 42% and 92% of subjects in 10 ppm and 32 ppm dosed groups, respectively. This warrants further investigations for additional critical parameters, such as effect on the intestinal epithelium permeability, especially with long term exposure. Due to the high potential of gastrointestinal exposure to AgNPs health supplements, it is important to understand the potential adverse health effects that may occur due to the changes in the intestinal mucosal permeability [16, 17].

**Fig. 1:**
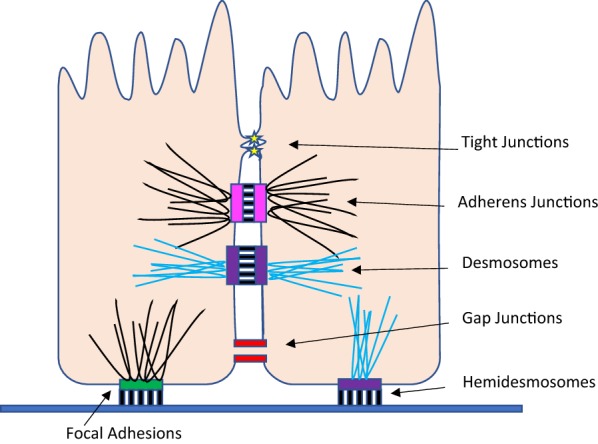
Six major types of cell junctions in the intestinal epithelium. (1) Tight junctions play a key role in sealing cells together and creating a barrier that is crucial in controlling paracellular transport. (2) Adherens junctions interact with the actin cytoskeleton and cadherins to support cell–cell adhesion. (3) Similarly, desmosomes adhere neighboring cells with cadherin proteins and intermediate filaments. (4) Gap junctions serve as cytoplasmic bridges between cells and are essential in communication and movement of small molecules. (5) Focal adhesions interact with integrins and actin filaments to promote adhesion between cells and the extra cellular matrix. (6) Finally, hemidesmosomes are also associated with integrins, but form connections with intermediate filaments in order to anchor cells to the extra cellular matrix. All six of these major junctions are central in epithelial adhesion, communication, and notably, intestinal permeability

The gastrointestinal tract (GIT) is the largest mucosal surface of the human body and is responsible for both barrier function, digestion of food, nutrients/water absorption, and excretion in healthy individuals. To carry these essential functions out properly, the intestinal epithelial cells (ICE) must maintain a constant state of homeostasis. Tight junctions, among other cell junctions, play a key role in sealing the intestinal epithelium to prevent harmful microbes and xenobiotics from entering systemic blood circulation [18]. A detailed schematic of the six major cell junction types is shown in Fig. 1. There are a wide variety of diseases and disorders associated with intestinal inflammation due to altered permeability including Crohn’s Disease, irritable bowel syndrome, and celiac disease [19, 20]. Importantly, oral exposure to AgNPs may cause alterations in intestinal permeability in healthy people or exacerbate the poorly regulated permeability in patients who suffer from gastrointestinal inflammation and other gastric disorders. Changes in the intestinal permeability may lead to exogeneous molecules cross the epithelial barrier, which can result in “leaky gut syndrome”, or nutrient deficiencies and malnutrition [21, 22]. On the other hand alteration in permeability may also activate immune cells leading to infection and inflammation [23]. Moreover, cytokines such as intestinal tumor necrosis factor α (TNF-α) are usually elevated during gastrointestinal inflammation [24].

In our previously published research, the 10 nm AgNPs were found to have the greatest impact on gut permeability, compared to other sizes of AgNPs, in an in vitro model [25]. In the present study, we utilized ileal tissue from Sprague–Dawley rats exposed to AgNPs by oral gavage to examine the effects on intestinal permeability via gene expression analysis. The purpose of this study was to compare our previous findings, where we used an in vitro intestinal epithelial cell culture model, to data derived from a 13-week oral gavage study in a rodent model to understand the potential alterations in intestinal permeability during AgNP exposure.

## Results

### Effect of AgNPs in male vs. female animals

**Fig. 2:**
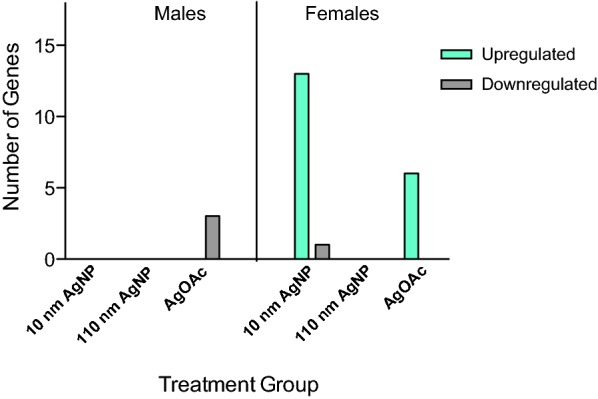
Comparative analysis of mRNA expression of genes involved in cell junctions during AgNP exposure. Number of genes upregulated and/or downregulated in males vs females treated with either AgNPs (10 nm or 110 nm) or AgOAc as compared to water/0.1% MC gavaged rats or 2 mM sodium citrate/0.1% CMC, respectively. Left panel shows the data obtained from male and the right panel shows the data obtained from female animals (n = 3 for each sex)

AgNPs (10 nm and 110 nm) and silver acetate (AgOAc) were suspended in sodium citrate and water, respectively. Carboxymethylcellulose (CMC) at 0.1% was used in the sodium citrate and all AgNP solutions, whereas 0.1% methylcellulose (MC) was used in the water and silver acetate solutions as vehicle to prevent the solutions from passing through the intestine too quickly. The AgOAc control in the study served to differentiate gene expression changes due to silver ions or the nanoparticles, specifically. After RNA extraction and cDNA synthesis, qPCR analysis was performed to assess the gene expression of cell junction and permeability genes in the small intestine. Interestingly, the numbers of genes upregulated and/or downregulated were entirely different in male and female animals (Fig. 2). Details regarding the fold change of each gene are represented in subsequent figures.

**Fig. 3:**
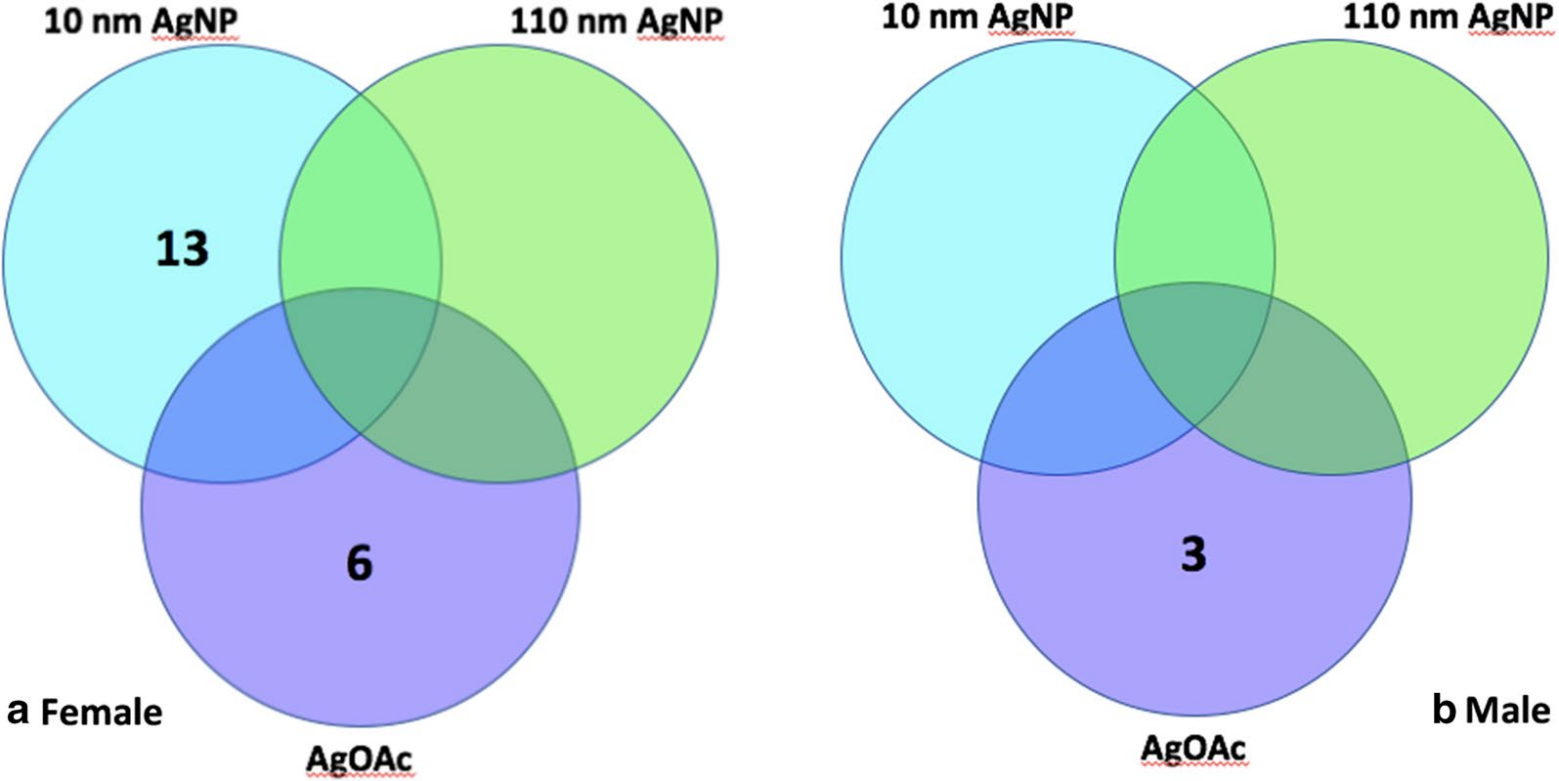
Perturbation of unique gene set (mRNA expression) in male and female animals during AgNP exposure. Venn diagram analysis of number of genes expressed in animals treated with either 10 nm AgNP, 110 nm AgNP, or AgOAc. **a** Number of genes upregulated in females. **b** Number of genes downregulated in males

Males experienced downregulation of several genes only in the AgOAc positive control (Fig. 3a). Three genes, Caveolin 1 (*Cav1*), Integrin alpha 8 (*Itga8*), and Occludin (*Ocln*) were downregulated in this group alone (Fig. 3a). On the other hand, female animals experienced upregulation of many genes in the 10 nm AgNP group and the AgOAc group compared to the control. In Venn diagram analysis, it was revealed that in females, 13 genes were upregulated only in the 10 nm AgNP group and 6 different genes were upregulated only in the AgOAc group. It was surprising that these changes were mutually exclusive across treatment groups. The 13 genes upregulated in the 10 nm AgNP group were Caveolin 1 (*Cav1*), Caveolin 2 (*Cav2*), Claudin 1 (*Cldn1*), Claudin 10 (*Cldn10*), Claudin 5 (*Cldn5*), Claudin 6 (*Cldn6*), Desmoglein 4 (*Dsg4*), Intracellular adhesion molecule 1 (*Icam1*), Integrin beta 2 (*Itgb2*), Notch homolog 1 (*Notch1*), Notch homolog 2 (*Notch2*), Platelet/endothelial cell adhesion molecule 1 (*Pecam1*), and Poliovirus receptor-related 1 (*Pvrl1*). The 6 genes upregulated in the AgOAc group were Claudin 15 (*Cldn15*), Claudin 9 (*Cldn9*), Gap junction protein gamma 3 (*Gja3*), Integrin alpha L *(Itgal*), Notch homolog 3 (*Notch3*), and Notch homolog 4 (*Notch4*).

The expression of a gene was considered upregulated or downregulated if the fold change was equal to or greater than twofold. However, changes in gene expression were only considered statistically significant with p ≤ 0.05.

### Changes in expression of tight junction genes

**Fig. 4:**
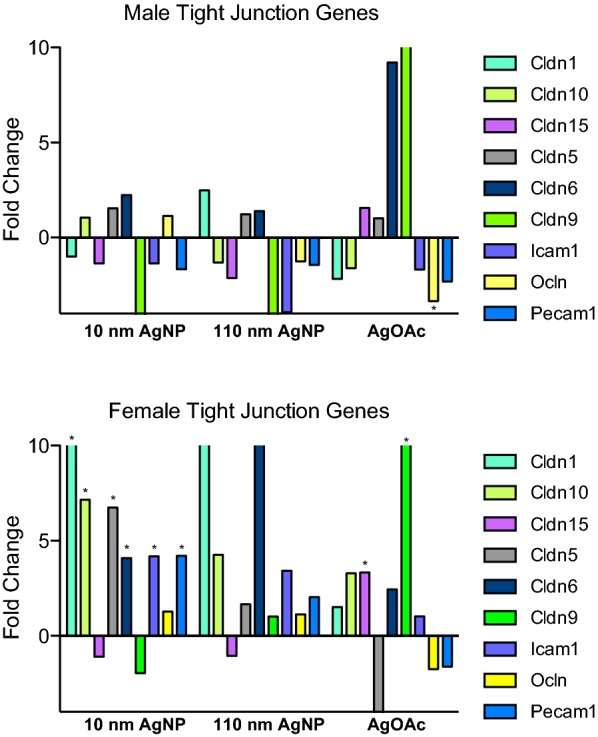
Differential expression of mRNA genes involved in maintenance of IEC tight Junction during AgNP exposure. Fold change during the treatment with AgNPs (10 nm and 110 nm) and AgOAc as compared to water/0.1% MC gavaged rats or 2 mM sodium citrate/0.1% CMC, respectively. Upper panel shows the fold change for male and the lower panel shows the fold change for female animals. These data represent an average of three animals in each group. The statistical significance (as compared to respective control) is indicated by * (p ≤ 0.05)

In males treated with AgNPs, there were fluctuations in tight junction genes, albeit they can be accounted as noise due to a lack of statistical significance (Fig. 4a). However, one gene, *Ocln*, was significantly downregulated in the AgOAc group.

In females, many of the claudin and other tight junction genes were upregulated after AgNP exposure (Fig. 4b). *Cldn1*, *Cldn10*, *Cldn5*, *Cldn6*, *Icam1*, and *Pecam1* were tight junction genes upregulated in only the 10 nm AgNP group. Some of these genes were upregulated with a greater magnitude than the others. Namely, *Cldn1*, *Cldn10*, and *Cldn5* were all significantly upregulated more than fivefold. In contrast, 2 different tight junction gene *Cldn15* and *Cldn9* were upregulated only in the AgOAc group. Overall, the tight junction genes were the most affected family of cell junction genes by AgNPs, especially in female animals.

### Changes in expression of focal adhesions

**Fig. 5:**
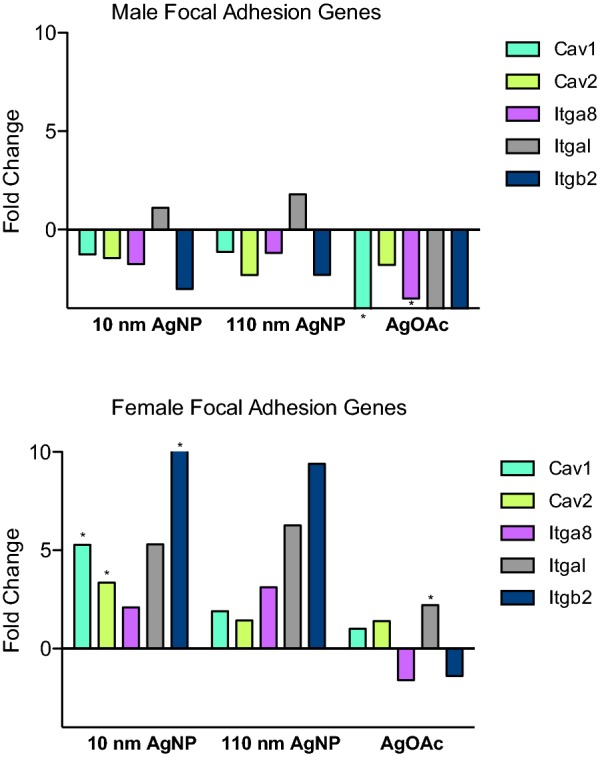
Differential expression of mRNA genes involved in IEC focal adhesion during AgNP exposure. Focal Adhesion genes fold change during the treatment with AgNPs (10 nm and 110 nm) and AgOAc as compared to water/0.1% MC gavaged rats or 2 mM sodium citrate/0.1% CMC, respectively. Upper panel shows the fold change for male and the lower panel shows the fold change for female animals. These data represent an average of three animals in each group. The statistical significance (as compared to respective control) is indicated by * (p ≤ 0.05)

Upon analysis of focal adhesion gene expression in males, only the group treated with AgOAc had a downregulation in *Cav1* and *Itga8* (Fig. 5a). Interestingly, females had a distinctly opposite pattern of focal adhesion gene expression (Fig. 5b). The group treated with 10 nm AgNP experienced a significant upregulation in *Cav1*, *Cav2*, and *Itgb2*, while the AgOAc group underwent upregulation of *Itgal*.

### Changes in expression of adherens junctions

**Fig. 6:**
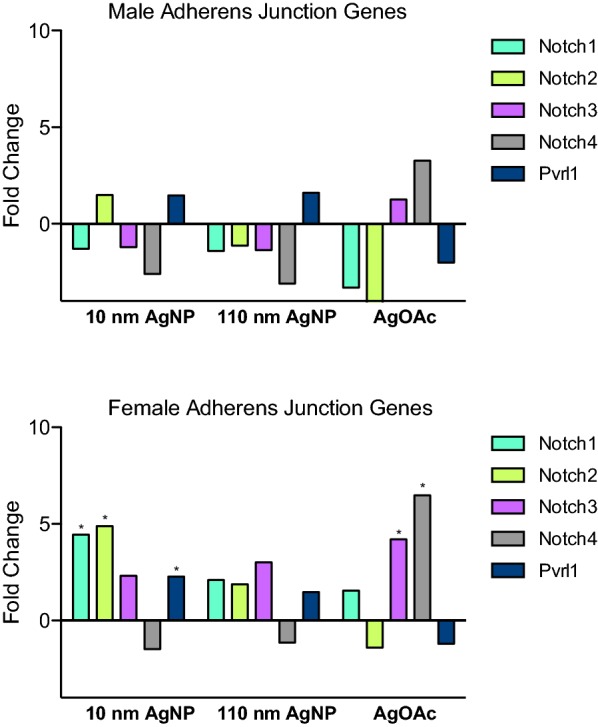
Differential expression of mRNA genes involved in IEC adherens junction during AgNP exposure. Adherens Junction genes fold change during the treatment with AgNPs (10 nm and 110 nm) and AgOAc as compared to water/0.1% MC gavaged rats or 2 mM sodium citrate/0.1% CMC, respectively. Upper panel shows the fold change for male and the lower panel shows the fold change for female animals. These data represent an average of three animals in each group. The statistical significance (as compared to respective control) is indicated by * (p ≤ 0.05)

Male rats did not experience significant alterations in adherens junction gene expression with AgNP treatment (Fig. 6a). When analyzing adherens junction genes in females, it was noted that *Notch1*, *Notch2*, and *Pvrl1* were upregulated in the 10 nm AgNP group (Fig. 6b). Interestingly, *Notch3* and *Notch4* were upregulated significantly in only the AgOAc treated group.

### Changes in expression of gap junctions

**Fig. 7:**
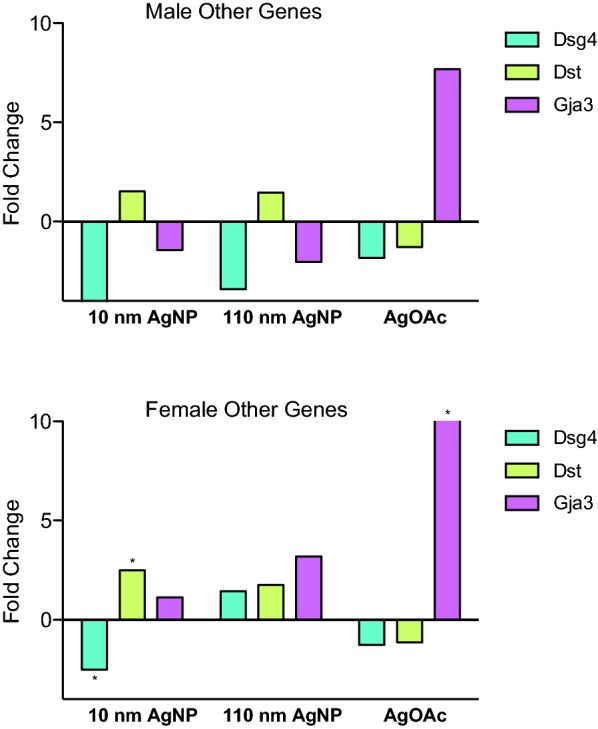
Differential expression of mRNA genes involved in intestine mucosa integrity during AgNP exposure. Fold change of *Dsg4, Dst*, and *Gja3* gene during the treatment with AgNPs (10 nm and 110 nm) and AgOAc as compared to water/0.1% MC gavaged rats or 2 mM sodium citrate/0.1% CMC, respectively. Upper panel shows the fold change for male and the lower panel shows the fold change for female animals. These data represent an average of three animals in each group. The statistical significance (as compared to respective control) is indicated by * (p ≤ 0.05)

Gap junction gene expression was not affected by AgNPs in male rats. In female animals, *Gja3* expression increased only in the AgOAc group, and this change was greater than tenfold (Fig. 7b).

### Changes in expression of desmosomes and hemidesmosomes

Male animals did not experience any changes in hemidesmosome genes, but female animals treated with 10 nm AgNP had a significant upregulation of Dystonin (*Dst*) (Fig. 7a, b). Furthermore, downregulation of *Dsg4*, a desmosome gene, was observed in female rats treated with 10 nm AgNP. Interestingly, this is the only downregulated gene observed in any female group of this project.

### Changes in protein level of TNF-α

**Fig. 8:**
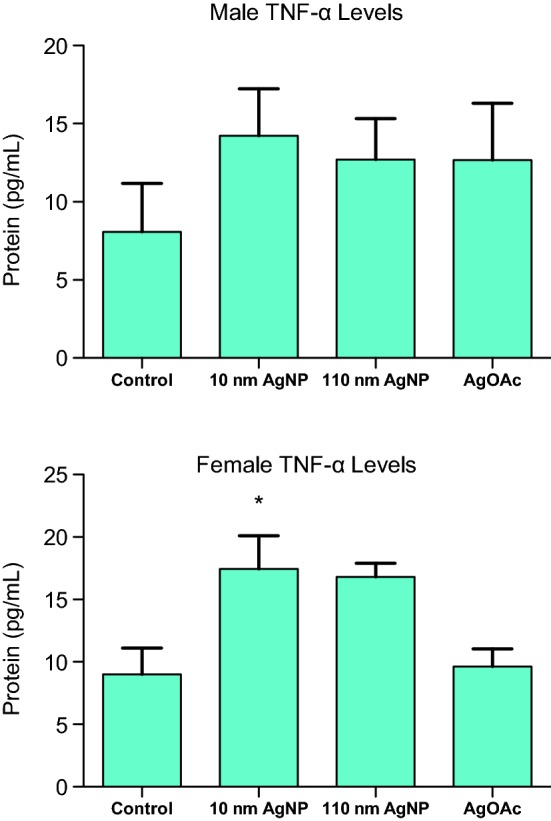
Secretion of TNF- α in the intestinal mucosa during the treatment with AgNP. Quantification of TNF-α during the treatment with 9 mg/kg BW AgNPs (10 nm and 110 nm) and AgOAc compared to water/0.1% MC gavaged rats or 2 mM sodium citrate/0.1% CMC, respectively. Upper panel shows the TNF-α level (in pg/ml) for male and the lower panel shows the TNF-α level (in pg/ml) for female animals. These data represent an average of three animals in each group. The statistical significance (as compared to respective control) is indicated by * (p ≤ 0.05)

AgNP caused an increased level of pro-inflammatory response (TNF-α secretion) in all experimental animals as compared to respective controls. However, male animals did not show statistically significant difference in the TNF-α secretion. Female animals treated with 10 nm AgNP had a significantly higher level of TNF-α (Fig. 8a, b).

## Discussion

The increased use of AgNPs has prompted the urgency to address the knowledge-gap regarding the potential gastrointestinal effects of AgNP exposure [26]. The structural integrity and barrier function of intestinal epithelial cells are regulated by several genes that include notch receptors, claudins, and desmosomes. These genes play a significant role in activating cell signaling for immune activation and mucin secretions to maintain barrier function. Furthermore, the single cell layer of intestinal epithelium plays an essential role in both nutrient absorption and barrier function in healthy individuals. Importantly, cell junctions, such as tight junctions, adherens junctions, and gap junctions, are held responsible for cell adhesion and communication within the intestinal epithelium [27–29]. AgNPs can interact with the host mucosa as nanoparticles, as well as, released ions or changed composition (e.g., to AgCl) in the stomach. The present study was designed to examine the changes in the gastrointestinal epithelial layer cell junction gene expression in male and female rats exposed orally to different sizes of AgNPs.

The results from this study indicate that there is a substantial difference of gene expression between male and female animals. In general, male animals experienced downregulation of cell junction genes, while female animals underwent upregulation, and many of those changes were statistically significant (Table 1). In females, 5 out of the 6 groups of cell junction genes were affected by 10 nm AgNPs. Tight junction (*Cldn1, Cldn5, Cldn6*, *Cldn10* and *Pecam1*), focal adhesion (*Cav1, Cav2*, and *Itgb2*), adherens junction (*Pvrl1, Notch1*, and *Notch2*), and hemidesmosome (*Dst*) groups were all upregulated significantly in females treated with 10 nm AgNP, indicating potential changes in intestinal permeability. It was also observed that most of the changes in female gene expression were in the tight junction family, specifically claudin genes. These genes have been studied thoroughly and the altered claudin genes in this study were noted to be classified as the “classic claudin” family [30]. Tight junctions are the most important junctions in the intestinal epithelium for the control of paracellular transport [18]. Specifically, *Cldn10* contributes to the formation of pore to facilitate paracellular transport.

**Table 1:** Summary of all differentially regulated genes involved in the maintenance of intestinal epithelial cells integrity

Gene	Family	Females			Males		
		10 nm AgNP	110 nm AgNP	AgOAc	10 nm AgNP	110 nm AgNP	AgOAc
*Cav1*	Focal adhesions	**↑**					**↓**
*Cav2*	Focal adhesions	**↑**					
*Cldn1*	Tight junctions	**↑**					
*Cldn10*	Tight junctions	**↑**					
*Cldn15*	Tight junctions			**↑**			
*Cldn5*	Tight junctions	**↑**					
*Cldn6*	Tight junctions	**↑**					
*Cldn9*	Tight junctions			**↑**			
*Dsg4*	Desmosomes	**↓**					
*Dst*	Hemidesmosomes	**↑**					
*Gja3*	Gap junctions			**↑**			
*Icam1*	Tight junctions	**↑**					
*Itga8*	Focal adhesions						**↓**
*Itgal*	Focal adhesions			**↑**			
*Itgb2*	Focal adhesions	**↑**					
*Notch1*	Adherens junctions	**↑**					
*Notch2*	Adherens junctions	**↑**					
*Notch3*	Adherens junctions			**↑**			
*Notch4*	Adherens junctions			**↑**			
*Ocln*	Tight junctions						**↓**
*Pecam1*	Tight junctions	**↑**					
*Pvrl1*	Adherens junctions	**↑**					

This table shows significant (p ≤ 0.05) alterations across all treatment groups as compared to its respective controls

As mentioned earlier, females exhibited greater changes in gene expression than males. This unambiguous difference between the sexes may be explained by hormonal physiology. Tight junctions are strictly regulated by sex hormones [31, 32]. Several of the genes that this study found to be altered significantly, such as *Pvrl1*, have been associated with progesterone regulation [33]. Additionally, the expression of *Cav1* has been linked to estrogen levels in rats [34, 35]. Remarkably, sexual dimorphism in response to exogenous substances has been found to be increasingly important in toxicological studies [16, 36]. Thus, it may be advantageous to monitor hormone levels in future in vivo studies, specifically with regard to the female menstrual cycle.

In intestinal epithelial cells, *Notch* signaling is involved in cell–cell communication with neighboring cells, and cross talk through *Wnt* signaling pathways of intestinal secretory cells [37]. *Notch* signaling is also responsible for differentiation of proliferated cells into goblet cells [38, 39], which is essential for secretion of secretary mucins. Additionally, a desmosome gene (*Dsg4*) was downregulated significantly in females treated with 10 nm AgNP. Since desmosomes are responsible for cell to cell adhesion in epithelial cells, these results suggest a loss of integrity in the intestinal epithelium.

The central goal of this study was to understand the impact of different sizes of nanoparticles on the permeability of the gastrointestinal system in male and females. Size differences between AgNPs and the release of ions from AgOAc may affect cellular components disparately, eliciting different gene expression patterns. We have previously shown higher microbicidal activity of smaller size AgNP (10 nm) as compared to larger size AgNP (110 nm) when animals were orally gavaged [17]. This difference was attributed to greater production of silver ions by 10 nm AgNP due to high surface area to volume ratio, suggesting it can exert more toxicity than a larger particle could. Moreover, the larger size AgNP (110 nm) may have tendency to agglomerate [17]. It is well known that commensal bacteria form a protective layer and maintains intestinal epithelial cell permeability. In vitro studies by our group [25] showed that the smaller nanoparticles are more capable of passing through cell junctions and disrupting essential processes. Thus, upregulation of the permeability related genes may be a defense mechanism by the host to protect itself.

In males, expression of some genes in tight junction (*Ocln*) and focal adhesion (*Itga8* and *Cav1*) groups was altered due to the exposure of AgOAc, but not AgNPs. Genes that were observed to have a decrease in expression, indicate looser cell junctions and an increase in intestinal permeability. Thus, it is tempting to speculate that silver ions (release via AgOAc) may have impact on the permeability in male rats, however, AgNPs did not have a significant effect on gene expression in male animals. Females also experienced changes in the expression of mRNA gene in the AgOAc group, albeit in different genes (*Cldn15*, *Cldn9*, *Gja3*, *Itgal*, *Notch3*, *Notch4*). One animal study revealed that *Cldn15* is critical for transporting Na^+^ through para-cellular spaces to the intestinal lumen for maintaining the ionic balance, which in turn facilitates the efficient absorption of glucose and other nutrients from the intestinal fluid [40]. Higher expression of *Gja3* could contribute to formation of gap junctions between two adjunct cells to release the pressure due to higher absorbance of solute molecules.

In this study, the tight junction family is the most adversely affected by AgNP exposure. Increased expression of the tight junction genes in females correlated with the increased secretion of TNF-α by the intestinal tissue. TNF-α is a pro-inflammatory cytokine and affects epithelial permeability. Increased intestinal permeability may further promote the exposure to luminal content and trigger an immunological response and intestinal inflammation [24, 41]. It is possible that the genes expressed differently are attempting to compensate the irritated and/or inflamed intestinal epithelium [42]. Barrier function is a critical responsibility assigned to claudins [43] and thus, gastrointestinal infections could be of particular concern in AgNP exposure [44]. Alternatively, it is important to consider that the changes in the cell junction gene expression or permeability could potentially lead to malnutrition and nutrient deficiencies. A recent study found that mice with anorexia experienced alterations in genes controlling intestinal permeability [45]. Additionally, mice with a double knockout of *Cldn2* experienced defective paracellular Na^+^ and nutrient transport in gut and died from malnutrition [46], suggesting that alterations in only a few cell junction genes can make a lethal impact on individuals. However, the weight of the female animals used in this study did not change significantly throughout the study when gavaged with AgNP [16]. AgNP gavaged male animals showed some increase in the body weight, but this increase was not considered biologically relevant [16].

Overall, it is important to note that many of the examined cell junction genes were altered significantly in animals exposed to AgNPs. Similarly, the in vitro conclusions from this group’s previous publication indicate AgNP exposure may cause subtle alterations in cell junctions and intestinal permeability [25]. Earlier reports described the effect of AgNPs on the blood–brain barrier (BBB) permeability in rat model; where intravenous, intraperitoneal, or intracerebral administration of nanoparticles resulted in the BBB breakdown in vivo [47]. To the authors’ knowledge, this is the first time that intestinal permeability alterations from oral AgNP exposure have been studied in a rat model. From this study, it is proposed that due to the oral exposure to AgNP, a pro-inflammatory reaction is initiated and may lead to changes in intestinal permeability. A cascade of these reactions may facilitate direct exposure of luminal content to gut-associated mucosal response and could potentially lead to the development of gastrointestinal inflammation/disease and/or nutrient deficiencies. More research is necessary for a complete understanding of the gender-specific differences along with the physiological and functional outcomes.

## Methodology

### Animal study

The ileal tissues used for this research were taken from an earlier study that evaluated particulate and ionic forms of silver and particle size for differences in silver accumulation, distribution, morphology, and toxicity when administered daily by oral gavage to Sprague–Dawley rats for 13 weeks [16]. Test materials and dose formulations were characterized by transmission electron microscopy (TEM), dynamic light scattering, and inductively coupled mass spectrometry (ICP-MS) as described earlier [16]. Seven-week-old male and female Sprague–Dawley rats (10 rats per sex per group) were randomly assigned to treatment: AgNP (10 or 110 nm) at 9, 18, and 36 mg/kg body weight (bw); and silver acetate (AgOAc) at 100, 200, and 400 mg/kg bw; and controls. AgNPs (10 nm or 110 nm) or AgOAc were compared to 2 mM sodium citrate/0.1% CMC or water/0.1% MC gavaged rats, respectively. At termination, complete necropsies were conducted, histopathology, hematology, serum chemistry, micronuclei, and reproductive system analyses were performed, and silver accumulations and distributions were determined [16]. Rat ileum (2 cm section) was collected from each rat at necropsy to determine the effects of test materials on the intestinal microbiome and gut-associated immune responses [17]. We showed that exposure to 10 nm AgNP at the lowest dose (9 mg/kg bw/day) was most detrimental for intestinal microbial population and gut-associated immune responses [17]. Thus, for the present study mRNA expression of the permeability related genes and protein levels of TNF-α in the intestinal tissue were evaluated in the animals gavaged with the smallest size and the lowest dose [10 nm AgNP (9 mg/kg bw/day)]. The mRNA expression levels were further compared with the largest size of the same dose animals [110 nm AgNP (9 mg/kg bw/day)] and AgOAC (400 mg/kg bw/day). AgNPs (10 nm or 110 nm) or AgOAc were compared to 2 mM sodium citrate/0.1% CMC or water/0.1% MC gavaged rats, respectively and served as controls. Each experimental and control group consisted of three individual animals from both male and female. A detailed experimental protocol for RNA extraction is published earlier [17].

### RNA extraction and qPCR analysis

The ileal tissues from Sprague–Dawley rats were thawed, then RNA was extracted using Trizol reagent (Molecular Research Center, Cincinnati, OH). Using the Turbo DNA-free kit (Life Technologies, Carlsbad, CA, USA), RNA was treated to remove any DNA contamination and then quantified using the NanoDrop® ND-1000 (NanoDrop, Wilmington, DE). Clean RNA was reverse transcribed into cDNA with the Invitrogen SuperScript IV Vilo kit (ThermoFisher, Carslbad, CA, USA). cDNA was analyzed using the RT^2^ Profiler PCR Array Rat Cell Junction Pathway Finder (Qiagen, Valencia, CA, USA) plates in an ABI 7500 Real-Time PCR system (Life Technologies, Carlsbad, CA, USA). Amplification was conducted in the following manner: 95 °C for 10 min, followed by 40 cycles of 95 °C for 15 s and 60 °C for 1 min. In addition, melt curve analysis was performed to verify the purity of each product. Each plate examined 84 unique genes for one sample and three samples were analyzed for each experimental group.

mRNA Gene expression data analysis was performed using the Qiagen Data Analysis Center (https://www.qiagen.com/us/shop/genes-and-pathways/data-analysis-center-overview-page/). Data was normalized using following housekeeping genes: Beta actin (*β*-*Actin*), Beta-2 microglobulin (*β2M*), Hypoxanthine phosphoribosyltransferase 1 (*Hprt1*), and Ribosomal protein large P1 (*Rplp0*). These housekeeping genes are constitutively expressed in all cells and are considered a reliable control in intestinal epithelium. Treatment groups exposed to either 10 nm AgNP or 110 nm AgNP were compared to the control group treated only with 0.1% carboxymethylcellulose (CMC). Furthermore, animals exposed to AgOAc were compared to the 0.1% methylcellulose (MC) control group. Statistical analysis was completed with a Student’s t-test. A p value of < 0.05 was chosen a priori to signify statistical significance.

### Protein extraction and TNF-α measurement

Protein lysate from the intestine was prepared using a gentleMACS-dissociator (Miltenyi Biotec Inc. Auburn, CA) as described earlier [48]. Levels of TNF-α were measured in the intestinal tissue lysate using bead-based assay described by Gokulan and co-workers [48]. Statistical analysis for TNF-α was conducted to compare of difference in the treatment groups using the *Mann*–*Whitney test* and a p value < 0.05 was considered significant.

## Data Availability

Upon request data would be available.
